# Transcriptome and Metabolome Analyses Reveal Molecular Mechanisms Regulating Growth Traits in Large Yellow Croaker (*Larimichthys crocea*)

**DOI:** 10.3390/ijms26199473

**Published:** 2025-09-27

**Authors:** Jiayi Fang, Yabing Wang, Jianguang Qin, Guangde Qiao, Qiaozhen Ke, Bingfei Li, Xiaoshan Wang, Shengyu Liu, Shiming Peng

**Affiliations:** 1 State Key Laboratory of Mariculture Biobreeding and Sustainable Goods, East China Sea Fisheries Research Institute, Chinese Academy of Fishery Sciences, Shanghai 200090, Chinawangyabing@ecsf.ac.cn (Y.W.); qiaogd@ecsf.ac.cn (G.Q.); 13173353721@163.com (B.L.);; 2 College of Fisheries and Life Science, Shanghai Ocean University, Shanghai 201306, China; 3 College of Science and Engineering, Flinders University, Adelaide, SA 5001, Australia

**Keywords:** aquaculture, enrichment analysis, growth regulation, body weight, gender

## Abstract

The large yellow croaker (*Larimichthys crocea*) is an economically important marine fish in China, whose growth rate in aquaculture has yet to meet the industry’s demands. Understanding the mechanism underlying inter-individual growth differences will create a favorable condition for selective breeding. In combined transcriptome and metabolome analyses, this study collected muscle tissues from four groups of croakers categorized based on sex and growth rate: fast-growing males, slow-growing males, fast-growing females, and slow-growing females. We identified 2344 differentially expressed genes (DEGs) and 198 differentially expressed metabolites (DEMs). Three genes, *bpgm*, *mstnb*, and *mylpfb*, played a crucial role in the growth regulation of large yellow croaker. The pathway enrichment analysis showed that “Aminoacyl-tRNA biosynthesis”, “Alanine, aspartate and glutamate metabolism”, “Inositol phosphate metabolism” and “Retrograde endocannabinoid signaling” pathways were involved in growth regulation. This study provides new clues for future research on the molecular mechanisms of growth regulation in large yellow croaker and builds a theoretical basis for improving the growth quality of this species.

## 1. Introduction

A growing number of studies have investigated genetic traits in aquatic organisms using omics technologies. Multi-omics approaches in various fish species have linked enhanced growth to nutrient metabolism, energy production, and protein synthesis [1,2]. In this study, we explored the molecular mechanisms underlying individual growth variations in large yellow croaker (*Larimichthys crocea*). Integrated transcriptomic and metabolomic analyses were performed on muscle tissue to identify key genes, regulatory metabolites, and signaling pathways associated with growth heterogeneity.

Large yellow croaker (*Larimichthys crocea*) belongs to Sciaenidae, Perciformes, and Osteichthys [3]. This species is nutritious and has high economic value. Its production has increased yearly in the past two decades, making it an important marine fish for aquaculture in China [4].

In large yellow croaker, the enriched *insulin-like growth factor 1* (*igf1*), fibroblast growth factor 19 (*fgf19*), isocitrate dehydrogenase 1 (*idh1*), malic enzyme 1 (*me1*), and other differentially expressed genes (DEGs) can impact growth by influencing cell proliferation, metabolic rate, and immune regulation [5].

In large yellow croaker, the significant expression of catalase, fatty acid desaturase enzyme 2, fatty acid synthase variant X1, and other genes can regulate growth by affecting cell proliferation, metabolic activity, and immune function [5]. At the genetic linkage map level, there are some studies on the transcriptome sequencing of species in the large yellow croaker family and the construction of a high-density genetic map. Four common growth-related quantitative trait loci (QTLs) were identified at a linkage group level [6]. They provide the basis for identifying the genetic loci of marker-assisted selection for growth traits of large yellow croaker in genetic breeding. A genome-wide association study (GWAS) on a large yellow croaker population with extreme growth phenotypes has revealed significant associations for growth-related traits such as eviscerated weight and identified a few potential functional genes involved in growth regulation [7]. Most studies on the growth traits of large yellow croakers have focused on comparative analysis among individuals with asynchronous growth rates. Various omics techniques have identified growth-related functional genes, and genetic maps have been drawn to locate QTLs related to growth traits [8]. These findings have provided the necessary background for further study of the growth traits of large yellow croaker, but little is known about the differential gene expression of growth traits between genders in large yellow croaker.

Differential growth between male and female large yellow croakers has been reported in the growth-out period [9]. The four groups of fish samples for analysis, namely the fastest-growing male (FGM) and female (FGF) and the slowest-growing male (LGM) and female (LGF), were of different sizes and genders but shared the same genetic background and environmental conditions. These discoveries offer valuable insights into the mechanisms underlying growth regulation in large yellow croaker. The results of this study are of great value and are practical in the selective breeding of large yellow croaker for fast growth traits.

## 2. Results

### 2.1. Transcriptome Analysis

#### 2.1.1. Transcriptome Data Processing and Quality Control

A total of 634,798,504 raw reads were generated from the samples by sequencing, and the raw sequencing data were further filtered to obtain 621.09 M clean reads after removing low-quality reads.

**Figure 1:**
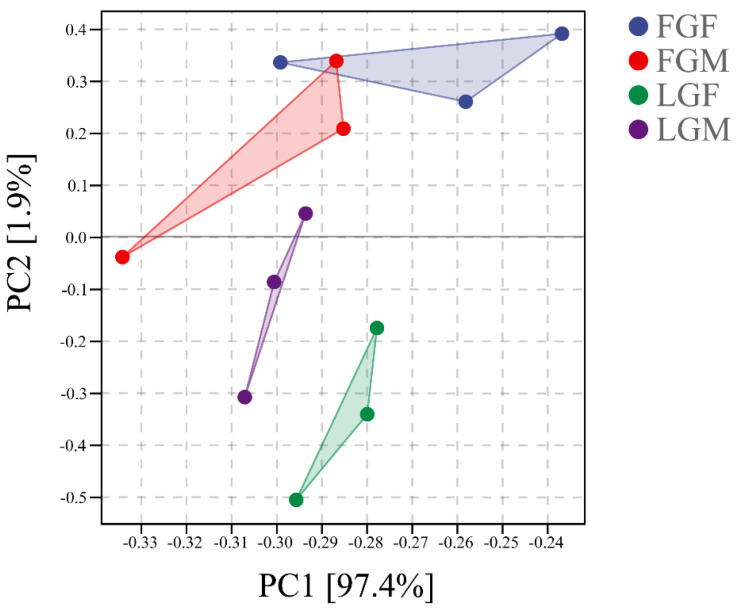
PCA for each group.

The unique mapping rate of filtered reads onto the large yellow croaker reference genome (GenBank accession: GCA_003845795.1) aligned with HISAT2 (v. 2.1.0, [10]) ranged from 90.14% to 92.29%, while the multi-mapping rate varied between 7.71% and 9.59% (Table S1). Hence, the transcriptome sequencing results were of high quality and met the requirements for follow-up work. Principal component analysis (PCA) showed a correlation between the samples (Figure 1), with a more pronounced separation between the four groups, which has implications for further research.

#### 2.1.2. DEGs Analysis

**Figure 2:**
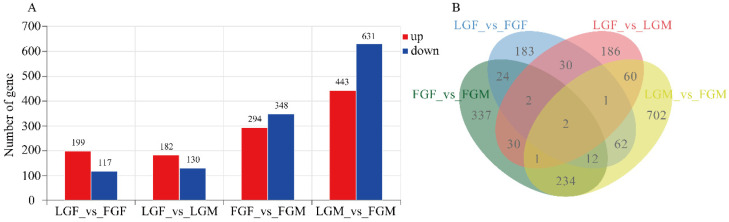
The number of DEGs depicted by (**A**) a bar chart and (**B**) a Venn diagram.

Using a 2 × 2 factorial design (factors: sex and growth), we identified DEGs through all possible pairwise comparisons between the four groups (FGF_vs_FGM, FGF_vs_LGF, FGM_vs_LGM, and LGF_vs_LGM). These DEGs were then analyzed in depth. We obtained 2344 DEGs, of which 1118 DEGs were up-regulated and 1226 DEGs were down-regulated (Figure 2). LGM_vs_FGM was enriched with the most DEGs (443 up-regulated and 631 down-regulated), followed by FGF_vs_FGM and LGF_vs_FGF, and LGF_vs_LGM was enriched with the fewest DEGs (182 up-regulated and 130 down-regulated), suggesting that there may be differences in genes regulating growth in males and females. The differences in gene expression between individuals with different growth rates were more significant in males than in females of large yellow croaker relative to females. Based on the analysis results, Figure 2 illustrates the number of DEGs across the comparison groups, as well as the overlap in gene expression among these groups(Table S2).

#### 2.1.3. Validation by qRT-PCR

**Figure 3:**
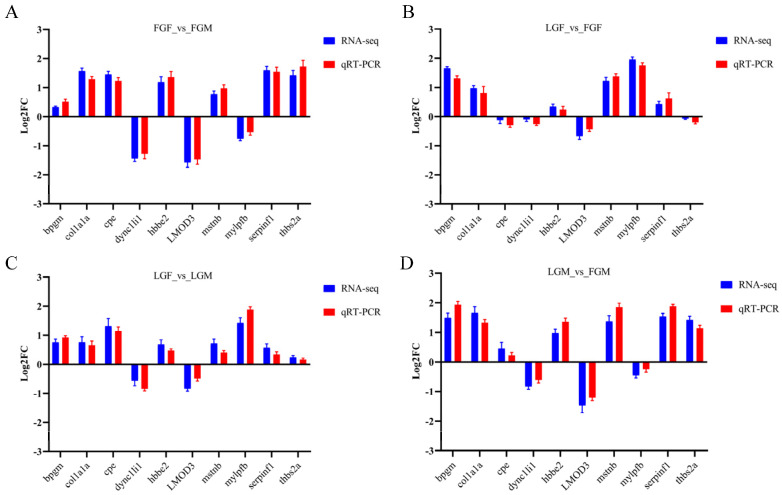
Comparison of relative expression levels measured by RNA-seq and qRT-PCR: (**A**) Comparison between FGF and FGM (FGF_vs_FGM); (**B**) Comparison between LGF and FGF (LGF_vs_FGF); (**C**) Comparison between LGF and LGM (LGF_vs_LGM); (**D**) Comparison between LGM and FGM (LGM_vs_FGM).

A total of ten DEGs (*bpgm*, *mstnb*, *mylpfb*, *LMOD3*, *COL1A1a*, *THBS2a*, *CPE*, *SERPINF1*, *HBBE2*, and *dync1li1*) were selected from the muscle transcriptome of large yellow croaker for validation. β-actin was used as the reference gene, and the primer sequences are listed in Table S4. The transcriptome data were validated using qRT-PCR. The results (Figure 3) confirmed that the expression patterns of these eight genes were consistent with the RNA-seq data.

### 2.2. Metabolome Analysis

#### 2.2.1. Multivariate Statistical Analysis

The results of PCA and orthogonal projections to latent structures discriminant analysis (OPLS-DA) score plots revealed that the metabolites were significantly different among different groups, with high repeatability between replicates (Figures S1 and S2).

#### 2.2.2. DEMs Analysis

**Figure 4:**
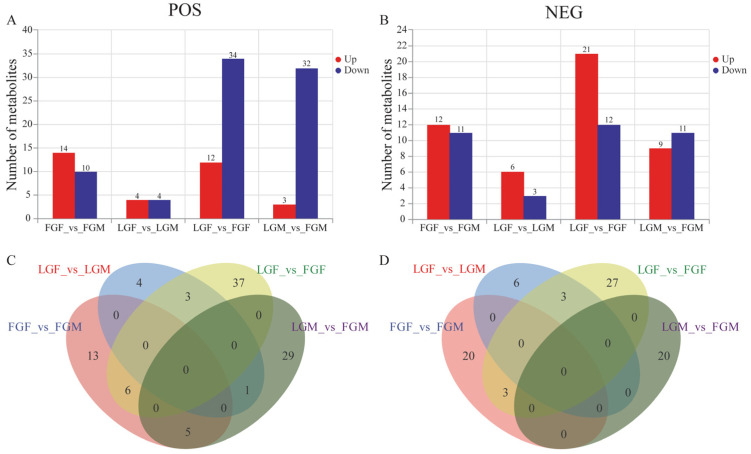
(**A**) Histogram of the number of DEMs screened for each group in the positive ion mode; (**B**) histogram of the number of DEMs screened for each group in the negative ion mode; (**C**) Venn diagram of DEMs in each group under positive ion mode; (**D**) Venn diagram of DEMs in each group under negative ion mode.

Based on the OPLS-DA model experiment results, Figure 4 displays the quantity of differentially expressed metabolites (DEMs) present in each comparison group. A total of 113 DEMs were identified in cationic mode, and 46 DEMs were identified in LGF_vs_ FGF (12 up-regulated and 34 down-regulated); 35 DEMs were selected from LGM_vs_FGM (3 up-regulated and 32 down-regulated); 24 DEMs were selected from FGF_vs_FGM (14 up-regulated and 10 down-regulated); and 8 DEMs were selected from LGF_vs_LGM (4 up-regulated and 4 down-regulated). In ion mode, 85 DEMs were identified overall. Among these, 33 DEMs were selected in LGF_vs_ FGF (21 up-regulated and 12 down-regulated); 20 DEMs were selected from LGM_vs_FGM (9 up-regulated and 11 down-regulated); 23 DEMs were selected from FGF_vs_FGM (12 up-regulated and 11 down-regulated); and 9 DEMs were selected from LGF_vs_LGM (6 up-regulated and 3 down-regulated). In both positive and negative ion modes, LGF_vs_ FGF enriched the most DEMs and LGF_vs_LGM enriched the least DEMs.

### 2.3. Metabolome and Transcriptome Association Analysis

#### 2.3.1. Correlation Analysis of Metabolites and Corresponding Transcripts

**Figure 5:**
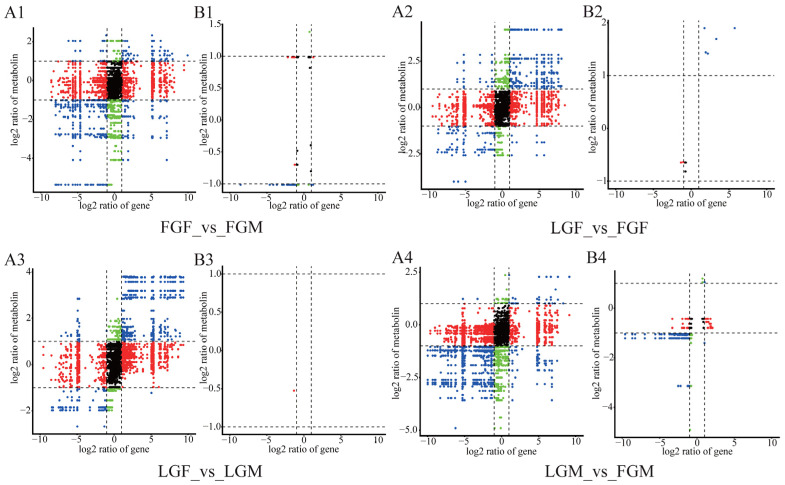
Correlation analysis between samples. (**A1**–**A4**) The nine quadrant plots of correlation analysis for each of the four groups. (**B1**–**B4**) Nine-quadrant plots of the results of DEG and DEM correlation analyses with coefficients above 0.8.

**Figure 6:**
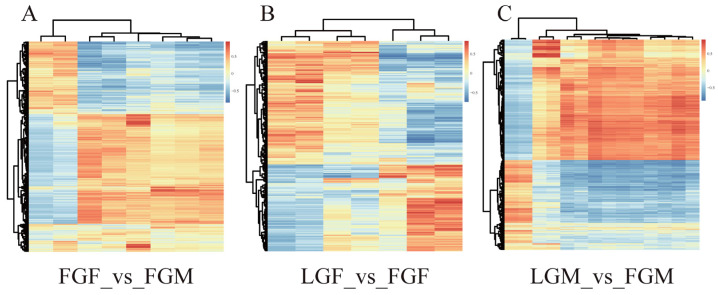
Differential gene and metabolite correlation analysis clustering heat maps with a correlation coefficient above 0.8: (**A**) FGF_vs_FGM; (**B**) LGF_vs_FGF; (**C**) LGM_vs_FGM.

A nine-quadrant plot was drawn to show the correlation of total genes and metabolites expressed in each group (Figure 5A1–A4). Correlation coefficients with Pearson’s correlation coefficient above 0.8 were selected to draw the difference multiple in the nine-quadrant diagram (Figure 5B1–B4). Based on these results, a clustering heat map of correlation coefficients was constructed (Figure 6A–C). Among them, only one DEM/DEG in LGF_vs_LGM had a Pearson correlation coefficient greater than 0.8 (Figure 5B3), so making heat maps for correlation analysis was unnecessary. The results indicated that the changing trend of DEGs in the comparison groups was similar to that of DEMs. The number of DEMs/DEGs with higher correlation in FGF_vs_FGM and LGM_vs_FGM exceeded that of the other two comparison groups.

#### 2.3.2. Differential Expression Results of Metabolites and Related Transcripts

**Figure 7:**
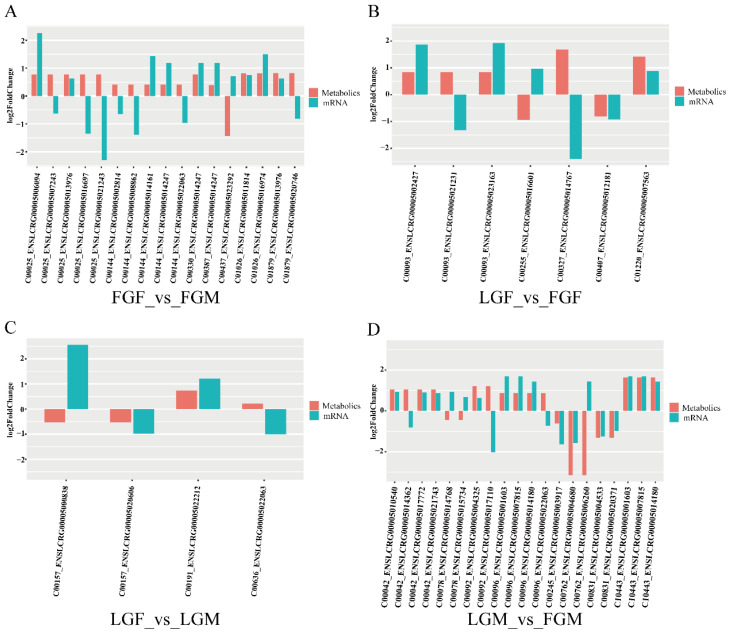
Differential expression results for metabolites and associated transcripts in each group: (**A**) FGF_vs_FGM; (**B**) LGF_vs_FGF; (**C**) LGF_vs_LGM; (**D**) LGM_vs_FGM.

We identified seventeen DEMs/DEGs in FGF_vs_FGM, among which nine DEMs/DEGs showed consistent expression trends, while eight DEMs/DEGs showed opposite trends. Seven DEMs/DEGs were identified in LGF_vs_FGF, among which four DEMs/DEGs showed consistent expression trends, while three DEMs/DEGs showed opposite trends. Four DEMs/DEGs were identified in LGF_vs_LGM, among which two DEMs/DEGs showed consistent expression trends, and two DEMs/DEGs showed opposite trends. We identified twenty DEMs/DEGs in LGM_vs_FGM, among which fourteen DEMs/DEGs showed consistent expression trends and six DEMs/DEGs showed opposite trends (Figure 7).

#### 2.3.3. Transcriptional and Metabolomic Co-Enrichment Pathways

A histogram was created to display the significance of concurrently enriched pathways based on the results of separate omics enrichment analyses of differential metabolites and genes (as shown in the Figure 7). LGF_vs_LGM had the fewest simultaneously enriched pathways. In contrast, FGF_vs_FGM and LGM_vs_FGM simultaneously enriched most of the pathways. From the *p*-value of the enrichment analysis of the two omics in the Figure 7, the differential co-enrichment pathways in each group can be visually seen. The co-differential pathways in FGF_vs_FGM included “Arginine biosynthesis” and “Glyoxylate and dicarboxylate metabolism”. The co-differential pathways in LGF_vs_FGF included “Alanine, aspartate and glutamate metabolism”, “Arginine biosynthesis”, and “Fatty acid degradation”. The pathways of “Glycine, serine and threonine metabolism” were co-differential in LGF_vs_LGM.2.3.4. Common Pathway Mapping of DEMs and DEGs

In order to understand the differential expression information of growth-related pathways in this study, the differentially expressed metabolites and all transcripts were associated and matched with the metabolic pathways and other related information in the KEGG database to obtain the common pathway information of DEG and DEM enrichment results in each comparison group. The analysis identified 28 pathways exhibiting significant differences in at least two comparison groups (Table 1), while 10 pathways showed significant differences across at least three. Further analysis showed that the pathways affecting the growth of large yellow croaker in both males and females include “Alanine, aspartate and glutamate metabolism”, “Aminoacyl-tRNA biosynthesis”, and “Thyroid hormone synthesis”, among others. These pathways and the differentially expressed information in the male and female groups a listed in Table S3, in which the up–down information of DEGs and DEMs in the pathway is expressed as “+/−”. The expression levels of DEGs/DEMs within the pathway are further illustrated in the Supplementary Material (Figure S3). These pathways were significantly enriched in common DEGs and different DEMs in both male and female groups, and these pathways played a vital role in the growth regulation of large yellow croaker.

Further analysis revealed that four metabolic pathways, including “Retrograde endocannabinoid signaling,” were consistently enriched in comparisons between genders within the same growth group (Table 2). The expression of DEGs/DEMs in the pathway is further demonstrated in the Supplementary Material (Figure S4). At the gene level, the gene UDP-glucuronosyltransferase-like was significantly up-regulated in the “Ascorbate and aldarate metabolism” pathway in both FGF_vs_FGM and LGF_vs_LGM, indicating that male fish with different growth rates had similar growth regulation pathways. No co-differential metabolites were found at the metabolic level, indicating that male and female individuals have different regulatory mechanisms. For the “Retrograde endocannabinoid signaling” pathway, the up-regulated expression of L-Glutamate enriched in FGF_vs_FGM and the down-regulated expression of Phosphatidylcholine enriched in LGF_vs_LGM were important regulatory factors affecting the growth of large yellow croaker. In addition, Pantothenate enriched in FGF_vs_FGM and Carnosine enriched in LGF_vs_LGM were significantly down-regulated in the “beta-Alanine metabolism” pathway. In the “Ascorbate and aldarate metabolism” pathway, D-Galactarate showed significant up-regulation in FGF_vs_FGM, while D-Glucuronate was significantly elevated in LGF_vs_LGM. Within the “Arachidonic acid metabolism” pathway, 5(S)-HETE was notably down-regulated in FGF_vs_FGM, and Phosphatidylcholine was considerably decreased in LGF_vs_LGM. These are important factors that affect the growth regulation of large yellow croaker males.

## 3. Discussion

In recent years, research on large yellow croaker using omics technology has involved many aspects in order to explore the mechanisms underlying functionality. Transcriptomics and metabolomics have been vital in studying disease resistance and immunity, growth and development, hypoxia tolerance, and low temperature and starvation stress of large yellow croaker. The successful application of these omics-based technologies will help clarify the potential molecular mechanisms behind the specific traits of large yellow croaker and offer theoretical support for cultivating this valuable species in the future.

In this research, the muscle tissues of large yellow croaker with different growth rates and genders were analyzed by transcriptomics and metabolomics. We screened 2344 DEGs (Figure 2) and 198 DEMs (Figure 4). Integrated analysis of transcriptomics and metabolomics data identified several growth-related pathways that exhibited common DEGs alongside distinct DEMs between male and female comparison groups (Table 1). Regardless of male and female individuals, the pathways involved in growth regulation are highly similar, and the same DEGs are enriched. However, the differentially expressed metabolites in males and females are slightly different. Within the same trait across different gender groups, several common gender-related metabolic pathways were significantly enriched (Table 2). No similar co-differential metabolites were found at the metabolic level, indicating that male and female individuals are slightly different in their regulatory mechanism. The combined analysis indicates that these pathways are crucial for regulating the growth of large yellow croaker.

**Figure 8:**
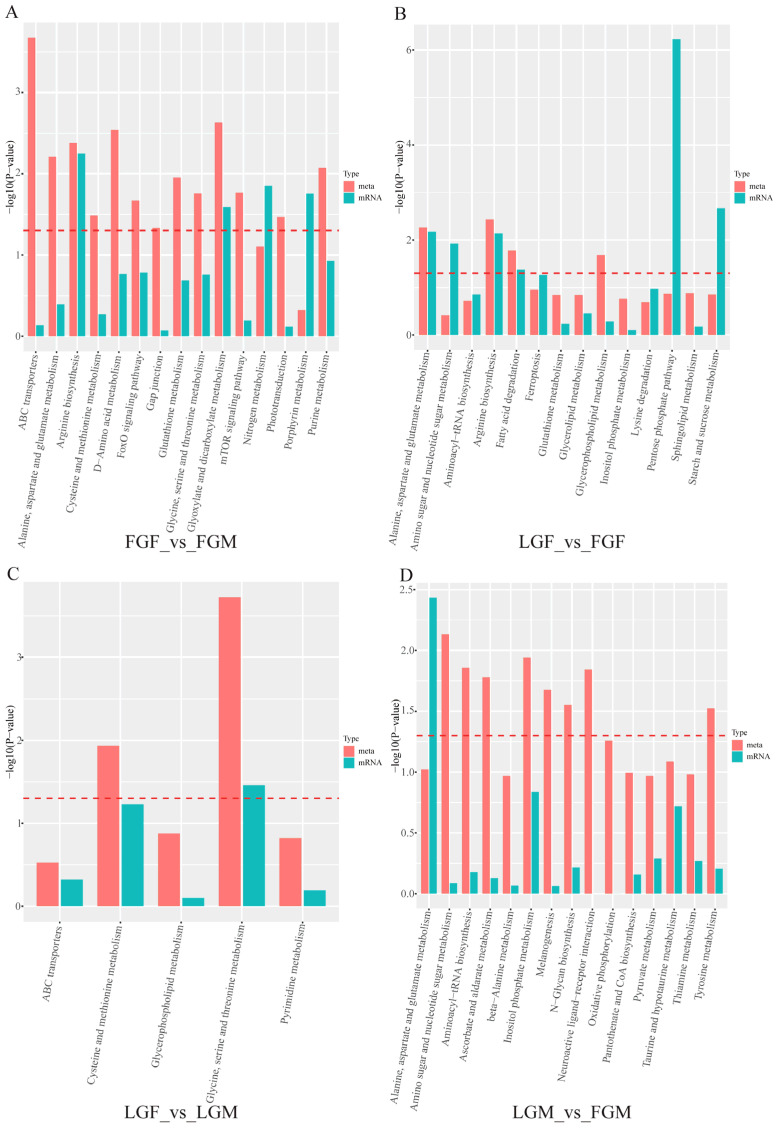
DEMs/DEGs co-enrichment KEGG pathway: (**A**) FGF_vs_FGM; (**B**) LGF_vs_FGF; (**C**) LGF_vs_LGM; (**D**) LGM_vs_FGM.

The number of DEGs in the male group was the highest, followed by the fast-growing groups of different genders (Figure 2). Both the fast-growing groups of different genders and the male groups with different growth rates had a greater number of highly correlated DEMs/DEGs, as well as the two co-enriched multi-omics pathways, than the other two control groups (Figure 5 and Figure 8). These results indicate that there may be differences in the genes and metabolites that regulate the growth of male and female croakers. The differences between male croakers with varying growth rates were significant, and the difference between male and female individuals in the fast-growing group was also significant.

The “Aminoacyl-tRNA biosynthesis” pathway is an essential metabolic process preceding amino acid biosynthesis and is closely associated with growth [11]. Aminoacyl-tRNA biosynthesis is closely related to protein digestion and absorption and is an essential metabolic pathway for protein synthesis and organism growth, suggesting that this pathway is closely related to growth [12,13]. In the male group within this study, two metabolites, L-Tyrosine and L-Tryptophan, were markedly down-regulated. Supplementing feed with tyrosine and phenylalanine can influence growth in juvenile fish, with phenylalanine as the sole precursor to tyrosine. Tyrosine, in turn, is a precursor for several molecules involved in controlling metabolism, growth, stress response, and pigmentation [14]. In another study, the addition of dietary Tryptophan (TRP) pronouncedly inhibited fish growth, which is also in line with the previous findings, suggesting that high expression of L-Tyrosine and L-Tryptophan affects the growth of large yellow croaker [15,16]. In contrast, significant down-regulation of L-Isoleucine was found in the female group. When the dietary concentration of Isoleucine is too high, protein synthesis decreases and catabolism increases, thus inhibiting protein deposition and fish growth [17]. Isoleucine promotes fish growth at optimal levels but inhibits it at higher doses [18,19]. These findings align with the results of the present study, suggesting that high expression of L-Isoleucine influences the growth of female large yellow croaker. Hence, the large yellow croaker can regulate individual growth through the pathway “Aminoacyl-tRNA biosynthesis”, but the amino acids involved in growth regulation differ in males and females.

Consistent with our observation of Alanyl-tRNA Synthetase (ASNS) down-regulation in enriched Ala/Asp/Glu metabolism, studies indicate that suppressing ASNS expression promotes growth in fish species [20,21]. The results of fish populations with different growth rates and genders suggest that the down-regulated expression of gene ASNS in the pathway “Alanine, aspartate and glutamate metabolism” can affect the growth of large yellow croaker regardless of female or male fish.

The co-enriched inositol phosphate metabolism pathway and up-regulated genes such as *tpi1b* are associated with enhanced protein synthesis and muscle growth in fish [22,23]. In this study, the gene tpi1b may indirectly stimulate protein synthesis by up-regulating its expression in the “Inositol phosphate metabolism” pathway, thereby further promoting the growth of large yellow croaker.

In the “Retrograde endocannabinoid signaling” pathway, endocannabinoids act as retrograde messengers at synaptic sites across various brain regions. Following depolarization or activation of receptors, these endocannabinoids are released from postsynaptic neurons and bind to CB1 receptors (CB1R) on presynaptic terminals. This binding inhibits Ca^2+^ channels, reducing the release of the inhibitory neurotransmitter GABA (depolarization-induced suppression of inhibition, DSI) or the excitatory neurotransmitter glutamate (depolarization-induced suppression of excitation, DSE). In addition to its well-known presence on the plasma membrane, CB1R is also found on mitochondrial membranes, where it diminishes mitochondrial respiration, further supporting DSI [24,25]. In this pathway, seven up-regulated and two down-regulated DEGs were enriched in LGF_vs_LGM and FGF_vs_FGM, respectively, and are associated with mitochondrial complex I (NADH:ubiquinone oxidoreductase) [26,27]. Complex I serves as the initial enzyme in the mitochondrial oxidative phosphorylation pathway [28]. Mitochondria, functioning as organelles within eukaryotic cells, are primarily responsible for generating cellular energy as ATP via oxidative phosphorylation. Therefore, the efficiency of complex I, a multi-subunit enzyme complex that is one of the components of the mitochondrial respiratory chain, is crucial for organisms to generate the energy they need [29].

At the level of metabolites, the metabolite L-Glutamate, enriched in FGF_vs_FGM, was significantly up-regulated in this pathway. Glutamic acid, or its ionic form L-Glutamate, is one of the most prevalent amino acids in nature, fulfilling essential roles at both the cellular and systemic levels [30]. In fish, Glutamate represents a significant portion of total amino acids, existing in both free and protein-bound states [31]. Dietary Glu supplementation has been found to boost intestinal antioxidant capacity, improve digestive and absorptive functions, and promote fish growth [32]. Combined with the results of this study, we have reason to speculate that the up-regulated expression of L-Glutamate in this pathway may promote growth by affecting the intestinal digestion and absorption capacity of male large yellow croaker.

In summary, we obtained evidence to differentiate a fast-growing male from a slow-growing male with metabolic pathways. The daily metabolism in the “Retrograde endocannabinoid signaling” pathway affects the activity of the multi-subunit enzyme complex complex I by encoding a series of mitochondrial genes. It affects mitochondrial function and the efficiency of oxidative phosphorylation, consequently affecting the growth of male croaker. The up-regulated expression of the metabolite L-Glutamate in this pathway may promote growth by influencing the digestive and absorptive functions in the intestine of male large yellow croaker.

## 4. Materials and Methods

### 4.1. Experimental Fish

In order to ensure a consistent genetic background and minimize the influence of extraneous variables on trait differentiation, the experimental fish used in this study were all selected from the “Fufa No. 1” breeding population of large yellow croaker (*Larimichthys crocea*). Specifically, more than 1200 individuals were obtained from a nursery farm in Ningde City, Fujian Province, China. The fish were then acclimated for 48 h under controlled conditions at the Fuding Research Centre of the East China Sea Fisheries Research Institute, Chinese Academy of Fishery Sciences, located in Fujian, China.

### 4.2. Experimental Design

The fish were first anesthetized in batches with a 20 mg/L eugenol solution, and the weight was measured using an electronic scale. The samples were classified into four intervals based on body weight data: fastest-growing females (FGF, 535.22 ± 47 g), fastest-growing males (FGM, 454.94 ± 61 g), slowest-growing females (LGF, 205.23 ± 21 g), and slowest-growing males (LGM, 198.30 ± 10 g). White muscle tissue was collected from six randomly selected fish per group (twenty-four fish in total). Within each group, the samples from every two fish were pooled to create one biological replicate, resulting in three pooled replicates per group (twelve samples in total). All pooled samples were immediately frozen in liquid nitrogen and stored at −80 °C until RNA and metabolite extraction.

### 4.3. Transcriptome Analysis

The RNA was extracted using TRIzol reagent (Invitrogen, Carlsbad, CA, USA) according to the manufacturer’s instructions. Sample integrity was then assessed with an Agilent 2100 bioanalyzer (Agilent Technologies, Inc., Santa Clara, CA, USA). Concentration and purity measurements of RNA were taken using an ND-2000 NanoDrop spectrophotometer (NanoDrop Technologies, Inc., Wilmington, DE, USA). High-quality RNA was reverse-transcribed to cDNA to construct cDNA libraries. These libraries were subjected to paired-end (PE) sequencing (2 × 150 bp) on an Illumina NextSeq 2000 platform. Raw sequencing data were quality-controlled and filtered using fastp (v0.23.4, [33]) with the following parameters: removal of adapter sequences, bases with a Phred quality score below Q30, and reads containing more than 5% undetermined bases (N). The resulting high-quality clean data were then aligned to the reference genome using HISAT2. Based on this alignment, gene expression levels were measured. Fragments per kilo base per million (FPKM) were adopted for normalization to compare gene expression levels across various genes and samples. PCA was performed for each sample based on expression levels using the DESeq software (https://www.r-project.org/ (accessed on 18 December 2024)) package in R language to assess the relevance of biological repetitive sequences. DEGs between groups were identified using the DESeq R package (version 1.38.0), applying criteria of *p*-value < 0.05 and log2(foldchange, FC) > 1 [34]. The DEGs sets in the differential expression analysis results of each group were statistically analyzed, and the DEGs with *p*-adjust < 0.05 and more significant differences were further enriched within the up-regulated and down-regulated ranges. Based on the differential analysis results, the unique differential genes shared between comparison groups were counted and presented in a Venn diagram. The common differential genes across each pairwise comparison group were also enriched and analyzed.

### 4.4. qRT-PCR Verification

RNAs (from muscle tissues) were subjected to real-time quantitative polymerase chain reaction (qRT-PCR) to validate the transcriptional expression levels of ten DEGs. Gene-specific primers were designed using Primer5 (Premier, Charlotte, NC, USA), with β-actin serving as the reference gene (primer sequences are provided in Table S4). Three biological replicates were performed.

qRT-PCR was carried out using a FastReal Rapid PCR Kit (Tiangen, Beijing, China). Amplification was performed in a 10 μL reaction volume containing 5 μL of 2× FastReal qPCR PreMix, 0.3 μL each of forward and reverse primers (10 μmol/L), 3.4 μL of ddH_2_O, and 1 μL of cDNA template. A two-step cycling protocol was adopted according to the manufacturer’s instructions. The thermal cycling conditions consisted of initial denaturation at 95 °C for 3 min, followed by 40 cycles of denaturation at 95 °C for 5 s, annealing at 60 °C for 15 s, and extension at 72 °C for 15 s. Melting curve analysis confirmed that each PCR product yielded a single peak. Data were analyzed using the 2^−ΔΔCt^ method.

### 4.5. Metabolomic Analysis

After samples were gradually thawed at 4 °C, a specified volume was combined with a methanol, acetonitrile, and water solution (2:2:1, *v*/*v*). Following vortex mixing and low-temperature sonication for 30 min, the samples were left at −20 °C for 10 min. They were centrifuged at 14,000 rpm for 20 min at 4 °C, and the supernatant was vacuum-dried. For mass spectrometry, reconstitution was achieved by adding 100 μL of aqueous acetonitrile (acetonitrile–water = 1:1, *v*/*v*), vortexed, and centrifuged at 14,000 rpm for 15 min at 4 °C. The resulting supernatant was separated on an Agilent 1290 Infinity LC ultra-high-performance liquid chromatography (UHPLC) HILIC column. The samples were analyzed in a randomized order to prevent the impact of instrumental drifts, with QC samples interspersed to monitor system stability and data reliability. Raw mass spectrometry data were converted into mzXML files using ProteoWizard MSConvert (v3.0, [35]) before being processed with XCMS (v3.18.0, [36]) for peak detection, alignment, and quantification. Metabolites were identified by matching precise mass and MS/MS spectra against standard databases (e.g., HMDB, KEGG). The processed data were subsequently imported into SIMCA (Version 14.0, Sartorius Stedim Data Analytics AB, Umeå, Sweden) for multivariate analysis, including unsupervised PCA and supervised OPLS-DA [34]. Biologically significant differential metabolite molecules were mined using strict “VIP > 1 and *p*-value < 0.05” as the screening criteria for DEMs.

### 4.6. Metabolome and Transcriptome Association Analysis

In the joint analysis, the data obtained from the previous transcriptome and metabolome analyses had to be unified and integrated to analyze the correlation between genes and metabolites detected in each differential group. Pearson’s correlation coefficients between genes and metabolites were determined using the cor function in R (https://www.r-project.org/ (accessed on 18 December 2024)), allowing for the assessment of correlations among all genes and metabolites expressed in each differential group. The screened DEGs and DEMs were subjected to further correlation analysis, and the coefficients with Pearson’s correlation coefficient of 0.8 or higher were selected and accordingly subjected to correlation coefficient clustering analysis. According to the differential analysis results of metabolome and transcript, the differential expression multiples of the differentially expressed metabolites and the corresponding differential transcripts with a *p*-value less than 0.05 in the differential analysis results were shown, and the differential expression results of metabolites and related transcripts were obtained. KEGG pathway analysis was conducted on enriched DEGs within each group. The KEGG analysis was conducted through enrichment analysis using the clusterProfiler package in the R software (version 3.6.1) (https://www.rproject.org/ (accessed on 21 December 2024)) [37]. Additionally, enriched DEMs were consolidated and annotated using the KEGG database (https://www.kegg.jp/ (accessed on 26 December 2024)) to elucidate their functions and interactions [38]. The results of the metabolic pathway analysis of important differential metabolites and associated transcripts were visualized using the R language pathview package (version 1.42.0).

## 5. Conclusions

We conducted comprehensive transcriptomic and metabolomic analyses on muscle tissues of large yellow croaker with varying growth rates and genders. Through joint analysis, DEGs were identified. These DEGs might be involved in growth. These findings revealed the molecular mechanisms underlying fish growth traits, providing valuable insights for breeding improved varieties. In summary, building on the multi-omics joint analysis paradigm, this study focused on large yellow croaker and links its growth regulation to specific molecular elements and mitochondrial energy metabolism, identifies sexual differences, and offers theoretical support for its differential aquaculture and breeding. However, it should be acknowledged that this study focused exclusively on muscle tissue and did not include other metabolic organs such as the liver, which may play significant roles in growth metabolism. Future research should incorporate multi-tissue omics approaches and functional validation experiments to further elucidate the systemic regulatory mechanisms of growth in non-muscle tissues.

## Figures and Tables

**Table 1:** Growth-related pathways and their differential expression information in male and female groups.

Pathway	DEGs		DEMs	
	LGF_vs_FGF	LGM_vs_FGM	LGF_vs_FGF	LGM_vs_FGM
Aminoacyl-tRNA biosynthesis	(−) arginyl-tRNA synthetase 1		(−) L-Isoleucine	(−) L-Tyrosine
	(−) asparaginyl-tRNA synthetase 1			(−) L-Tryptophan
Alanine, aspartate, and glutamate metabolism	(−) asparagine synthetase		(−) N-Acetyl-L-aspartate	(+) Succinate
	(−) argininosuccinate synthase 1		(+) D-Glucosamine 6-phosphate	
Inositol phosphate metabolism	(+) phospholipase C, epsilon 1		(+) 1D-myo-Inositol 1,4-bisphosphate	(+) D-Glucose 6-phosphate
	(+) triosephosphate isomerase 1b			
	(−) phospholipase C, epsilon 1			(−) D-Glucuronate
Central carbon metabolism in cancer	(+) phosphofructokinase, muscle b		(−) L-Leucine	(−) L-Leucine
	(+) lactate dehydrogenase A4			(+) D-Glucose 6-phosphate
	(+) phosphoglycerate mutase 2			(+) Succinate
Mineral absorption	(−) hephaestin like 1		(−) L-Isoleucine	(−) L-Tryptophan
Protein digestion and absorption	(−) solute carrier family 16 member 10		(−) L-Isoleucine	(−) L-Tyrosine
				(−) L-Tryptophan
Amino sugar and nucleotide sugar metabolism	(−) UDP-N-acetylglucosamine pyrophosphorylase 1, like 1		(+) D-Glucosamine 6-phosphate	(+) alpha-D-Galactose 1-phosphate
	(+) glucose-6-phosphate isomerase b			
	(+) phosphoglucomutase-1			(+) GDP-mannose
Thyroid hormone synthesis	(+) ATPase Na+/K+ transporting subunit beta 1b	(−) ATPase Na+/K+ transporting subunit beta 1b	(+) Glutathione disulfide	(+) D-Glucose 6-phosphate
Insulin resistance	(+) protein kinase, AMP-activated, gamma 3a non-catalytic subunit		(+) D-Glucosamine 6-phosphate	(+) D-Glucose 6-phosphate
Starch and sucrose metabolism	(+) glucose-6-phosphate isomerase b		(+) alpha-D-Glucose 1,6-bisphosphate	(+) D-Glucose 6-phosphate
	(+) phosphoglucomutase-1			
	(+) glycogen debranching enzyme			

**Table 2:** Gender-related pathways and their differential expression information in each group.

Pathway	DEGs		DEMs	
	FGF_vs_FGM	LGF_vs_LGM	FGF_vs_FGM	LGF_vs_LGM
Retrograde endocannabinoid signaling	(+) mitogen-activated protein kinase 8a	(+) NADH:ubiquinone oxidoreductase subunit B1	(+) L-Glutamate	(−) Phosphatidylcholine
		(+) NADH dehydrogenase subunit 1		
	(−) NADH:ubiquinone oxidoreductase subunit S5			
		(+) NADH dehydrogenase subunit 3		
	(+) fatty acid amide hydrolase			
		(+) NADH dehydrogenase subunit 4L		
	(+) calcium voltage-gated channel subunit alpha1 S			
		(+) NADH dehydrogenase subunit 4		
	(−) NADH:ubiquinone oxidoreductase core subunit S1			
		(+) NADH dehydrogenase subunit 5		
	(+) N-acyl phosphatidylethanolamine phospholipase D			
		(+) NADH dehydrogenase subunit 6		
beta-Alanine metabolism	(−) carnosine dipeptidase 2	(+) glutamate decarboxylase 1	(−) Pantothenate	(−) Carnosine
Ascorbate and aldarate metabolism	(+) UDP-glucuronosyltransferase	(+) UDP-glucuronosyltransferase	(+) D-Galactarate	(+) D-Glucuronate
	(−) UDP-glucose 6-dehydrogenase			
	(+) regucalcin			
Arachidonic acid metabolism	(+) gamma-glutamyltransferase 5a	-	(−) 5(S)-HETE	(−) Phosphatidylcholine
	(−) prostaglandin E synthase 3b (cytosolic)			

## Data Availability

Data is contained within the article and Supplementary Materials.

## Supplementary Materials

The following supporting information can be downloaded at: https://www.mdpi.com/article/10.3390/ijms26199473/s1.
