# Supplementary material for: Transcriptome and Metabolome Analyses Reveal Molecular Mechanisms Regulating Growth Traits in Large Yellow Croaker (Larimichthys crocea)
Source: Int J Mol Sci. 2025 Sep 27;26(19):9473. doi: 10.3390/ijms26199473 (PMC12525493; doi:10.3390/ijms26199473)
Supplement: Supplementary file 1 [file ijms-26-09473-s001.zip › Figure S1.pdf]

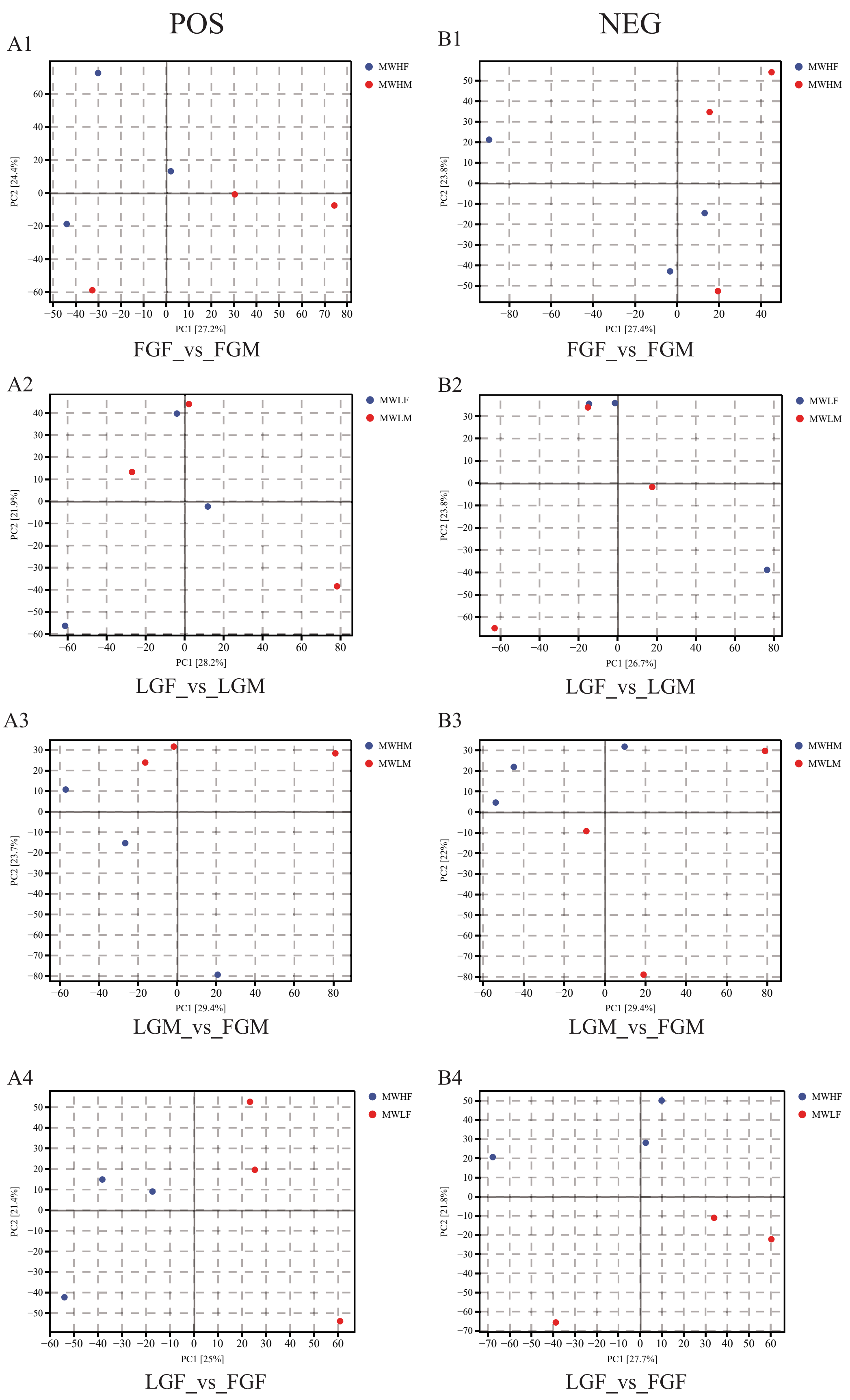

Figure S1. A1, A2, A3, A4 are the PCA score plots obtained in positive ion mode; B1, B2, B3, B4 are the PCA score plots obtained in negative ion mode
