# Supplementary material for: Transcriptome and Metabolome Analyses Reveal Molecular Mechanisms Regulating Growth Traits in Large Yellow Croaker (Larimichthys crocea)
Source: Int J Mol Sci. 2025 Sep 27;26(19):9473. doi: 10.3390/ijms26199473 (PMC12525493; doi:10.3390/ijms26199473)
Supplement: Supplementary file 1 [file ijms-26-09473-s001.zip › Figure S3.pdf]

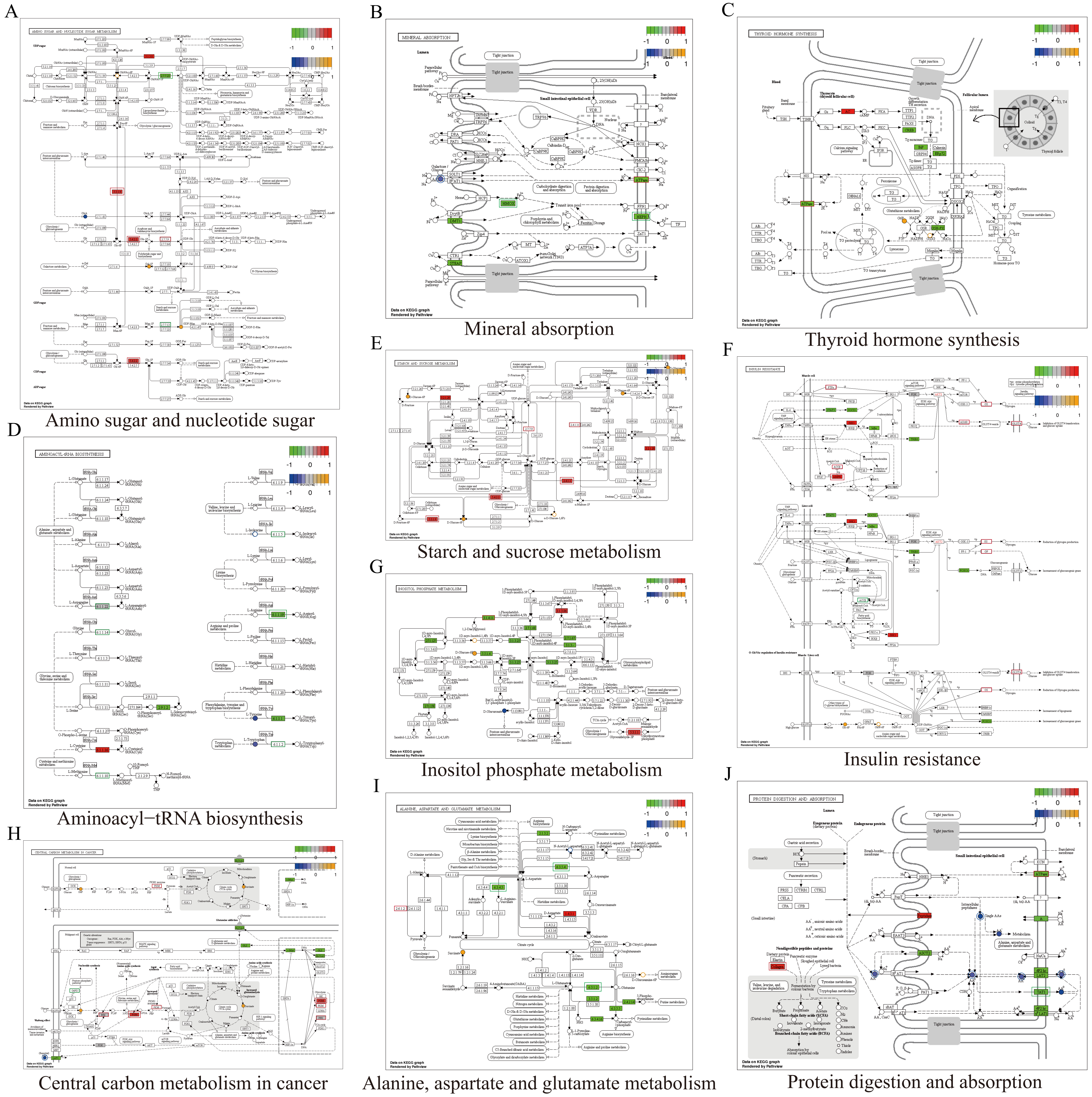

Figure S3. The growth-related pathways in KEGG were enriched in the male-female comparison group. Note: The circular node in the figure is the metabolite, and the square node is the enzyme corresponding to the transcript. The differential expression multiples of metabolites were represented from low to high in blue to yellow, and the differential expression multiples of transcripts were represented from low to high in green to red. The color in the box indicates the differential expression of the male fish group, and the border color indicates the differential expression of the female fish group.
