# Supplementary material for: Transcriptome and Metabolome Analyses Reveal Molecular Mechanisms Regulating Growth Traits in Large Yellow Croaker (Larimichthys crocea)
Source: Int J Mol Sci. 2025 Sep 27;26(19):9473. doi: 10.3390/ijms26199473 (PMC12525493; doi:10.3390/ijms26199473)
Supplement: Supplementary file 1 [file ijms-26-09473-s001.zip › Table S1.pdf]

Table S1 Summary of transcriptome data

Group	Raw Reads	Clean Reads	Total Mapped	Multiple Mapped	Unique Mapped
FGF_1	65,307,358	63,824,326	61,246,770 (95.96%)	5,474,826 (8.94%)	55,771,944 (91.06%)
FGF_2	64,504,216	63,035,248	60,630,432 (96.18%)	5,813,036 (9.59%)	54,817,396 (90.41%)
FGF_3	55,531,042	54,258,784	52,316,816 (96.42%)	4,689,109 (8.96%)	47,627,707 (91.04%)
FGM_1	57,421,202	56,110,028	54,115,089 (96.44%)	5,336,256 (9.86%)	48,778,833 (90.14%)
FGM_2	57,666,582	56,402,906	54,261,513 (96.20%)	5,157,328 (9.50%)	49,104,185 (90.50%)
FGM_3	57,224,062	55,921,748	53,792,659 (96.19%)	4,964,347 (9.23%)	48,828,312 (90.77%)
LGF_1	55,938,470	54,793,732	52,374,959 (95.59%)	4,350,794 (8.31%)	48,024,165 (91.69%)
LGF_2	48,676,682	47,640,508	45,536,339 (95.58%)	4,386,466 (9.63%)	41,149,873 (90.37%)
LGF_3	41,731,006	40,983,220	39,177,824 (95.59%)	3,563,186 (9.09%)	35,614,638 (90.91%)
LGM_1	43,837,130	42,906,766	41,088,549 (95.76%)	3,876,962 (9.44%)	37,211,587 (90.56%)
LGM_2	43,011,576	42,141,310	40,323,063 (95.69%)	3,107,648 (7.71%)	37,215,415 (92.29%)
LGM_3	43,949,178	43,067,518	41,243,075 (95.76%)	3,681,802 (8.93%)	37,561,273 (91.07%)
